# A bibliometric analysis of the recent advances in diazepam from 2012 to 2021

**DOI:** 10.3389/fphar.2022.1042594

**Published:** 2022-11-09

**Authors:** Ming Zhang, Lin Kou, Yaya Qin, Junwen Chen, Dazhang Bai, Li Zhao, Hongyu Lin, Guohui Jiang

**Affiliations:** Department of Neurology, Affiliated Hospital of North Sichuan Medical College, Institute of Neurological Diseases, North Sichuan Medical College, Nanchong, China

**Keywords:** diazepam, bibliometrics, visual analysis, Web of Science, CiteSpace, VOSviewer

## Abstract

**Background:** Diazepam is a classic benzodiazepine drug that has been widely used for disorders such as anxiety, sleep disorders, and epilepsy, over the past 59 years. The study of diazepam has always been an important research topic. However, there are few bibliometric analyses or systematic studies in this field. This study undertook bibliometric and visual analysis to ascertain the current status of diazepam research, and to identify research hotspots and trends in the past 10 years, to better understand future developments in basic and clinical research.

**Methods:** Articles and reviews of diazepam were retrieved from the Web of Science core collection. Using CiteSpace, VOSviewer, and Scimago Graphica software, countries, institutions, authors, journals, references, and keywords in the field were visually analyzed.

**Results:** A total of 3,870 publications were included. Diazepam-related literature had high volumes of publications and citations. The majority of publications were from the USA and China. The highest number of publications and co-citations, among the authors, was by James M Cook. Epilepsia and the Latin American Journal of Pharmacy were the journals with the most publications on diazepam and Epilepsia was the most frequently cited journal. Through a comprehensive analysis of keywords and references, we found that current research on diazepam has focused on its mechanism of action, application in disease, pharmacokinetics, risk, assessment, and management of use, status epilepticus, gamma-aminobutyric acid receptors (GABAR), intranasal formulation, gephyrin, and that ultra-performance liquid chromatography-tandem mass spectrometry (UPLC-MS/MS) is the current research hotspot.

**Conclusion:** Research on diazepam is flourishing. We identified research hotspots and trends in diazepam research using bibliometric and visual analytic methods. The clinical applications, mechanisms of action, pharmacokinetics, and assessment and management of the use of diazepam are the focus of current research and the development trend of future research.

## 1 Introduction

Diazepam has been one of the most successful and influential drugs from the psychopharmacological revolution since the 1950s. It is widely used for the treatment of insomnia, anxiety, epilepsy, pain, depression, muscle spasms, convulsions, alcohol withdrawal, and anesthesia induction. Furthermore, diazepam has become one of the best-selling drugs in history due to its extensive therapeutic range, rapid onset of action, reliable efficacy, high bioavailability, low toxicity, and low price (Calcaterra and Barrow, 2014). Although diazepam is the most popular and classical benzodiazepine, it still has adverse side effects such as drug tolerance, addiction, abuse, memory loss, withdrawal reactions, falls even death (Horowitz et al., 2021; Jeffery et al., 2019; Votaw et al., 2019). Moreover, the prescription of benzodiazepines has been severely restricted due to increasing attention to the risks of diazepam. However, its use is not falling but remains high (Agarwal and Landon, 2019). There was even a trend of increasing use during the COVID-19 pandemic (Campitelli et al., 2021; de Dios et al., 2021; Milani et al., 2021). To our knowledge, there has been a lack of quantitative and systematic analytical studies of the literature related to diazepam in the last decade.

Bibliometrics, first introduced by Alan Pritchard in 1969, as a method for statistical and quantitative analysis of scientific publications in a specific field and journal, is an excellent approach which combines with data visualization. As a result, it enables the mapping of knowledge structure clearly, presents recent development, and predicts trends in a given field (Pritchard, 1969; Samanta et al., 2022). It is currently used in many medical fields as an emerging method (Wilson et al., 2021). Herein, we used a bibliometric approach to quantitatively analyze the publications on diazepam from 2012 to 2022. This study was used to better understand the global research trends, the current status, hot spots, as well trends of diazepam, and to provide a reference and research direction for the subsequent application of diazepam.

## 2 Methods and research design

### 2.1 Data sources and collection

**FIGURE 1:**
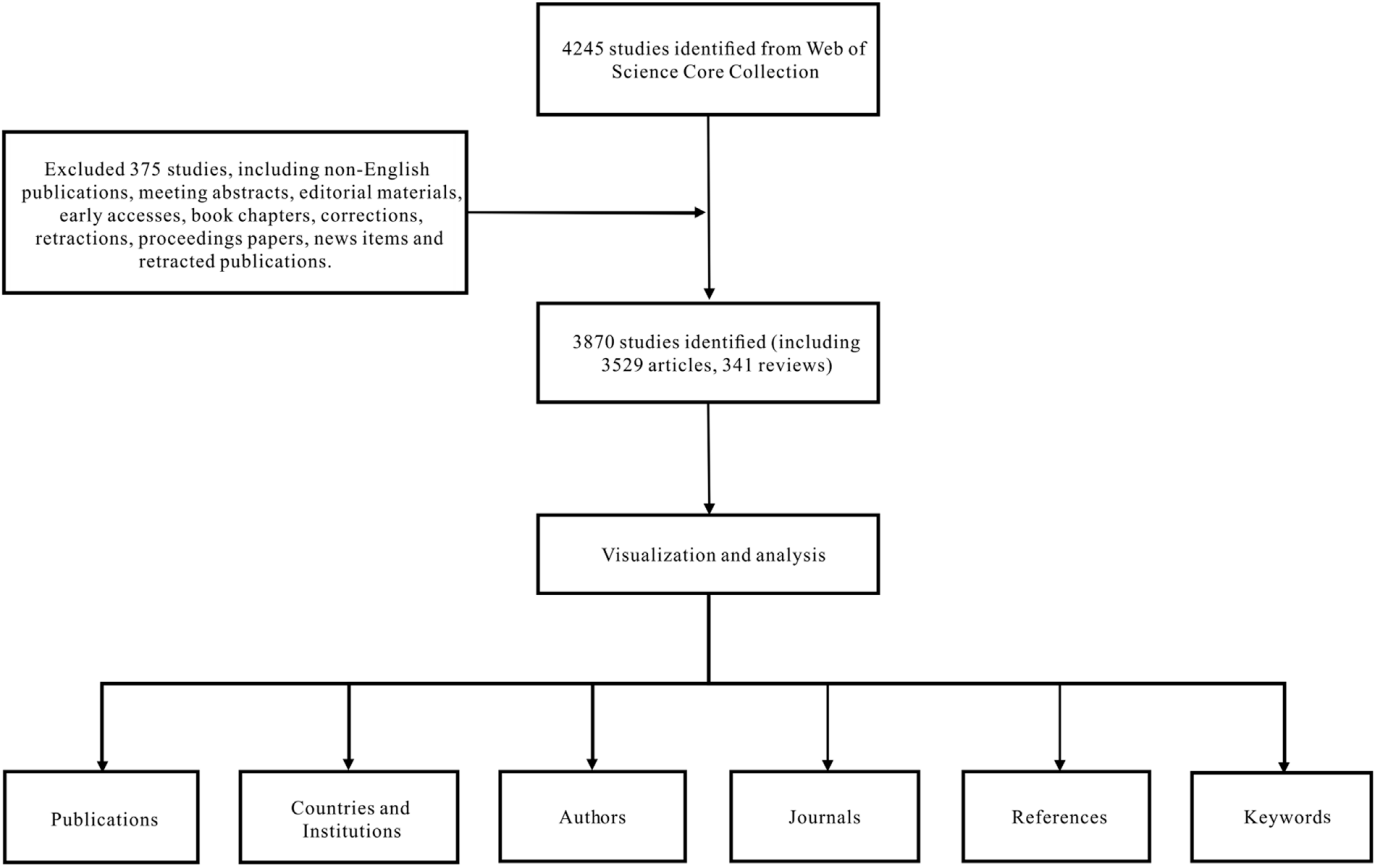
Research design of literature selection.

Data were extracted from the SCI-EXPANDED database of the Web of Science Core Collection and downloaded within a day on 10 July 2022. The search formula was set to TS = (diazepam), documents ranging from 1 January 2012 to 31 December 2021, were retrieved, and the language was limited to English. A total of 4,245 articles were retrieved and 375 irrelevant articles, including non-English publications, meeting abstracts, early accesses, editorial material, book chapters, retractions, corrections, proceedings papers, retracted publications, and news items were excluded. A total of 3,870 articles were exported in the form of records and references, and saved in a plain text file format (Figure 1).

### 2.2 Data analysis and tools

All data were collected in the Web of Science Core Collection and imported into VOSviewer, CiteSpace, and Scimago Graphica for visual analysis. VOSviewer is a sociometric network analysis software developed in 2009 at Leiden University. VOSviewer pays particular attention to the graphical representation of bibliometrics and has advantages in displaying large bibliographies. The purpose of VOSviewer is to illustrate bibliometric networks, construct visual network maps, and facilitate a comprehensive understanding of the structure and development of scientific research (van Eck and Waltman, 2010). CiteSpace is another visualization software for bibliometrics, developed by Professor Chen, based on sciometrics and data visualization technology. It can visually represent research hotspots and evolutionary processes and predict development trends in various fields. It is an effective way to analyze big data (Cheng et al., 2022).

Before analysis, all the data were thoroughly cleaned. Synonyms were classified into the same word, and meaningless keywords were deleted. Besides, a region of a country was attributed to that country, and different names of the same person/country/institution were merged. We not only used Microsoft Office Excel 2019 to analyze trends in the annual number of published articles and number of citations, but also used CiteSpace 6.1.R2 software to visualize references and timelines. Keywords were analyzed using VOSviewer. Data from VOSviewer were imported in Graph Modeling Language (GML) format into Scimago Graphica software to visually analyze countries, institutions, and authors. In addition, we obtained the journal impact factor (IF) and Journal Citation Reports (JCR) category quartile in 2021 from Web of Science.

## 3 Results

### 3.1 Annual outputs and citation trends

**FIGURE 2:**
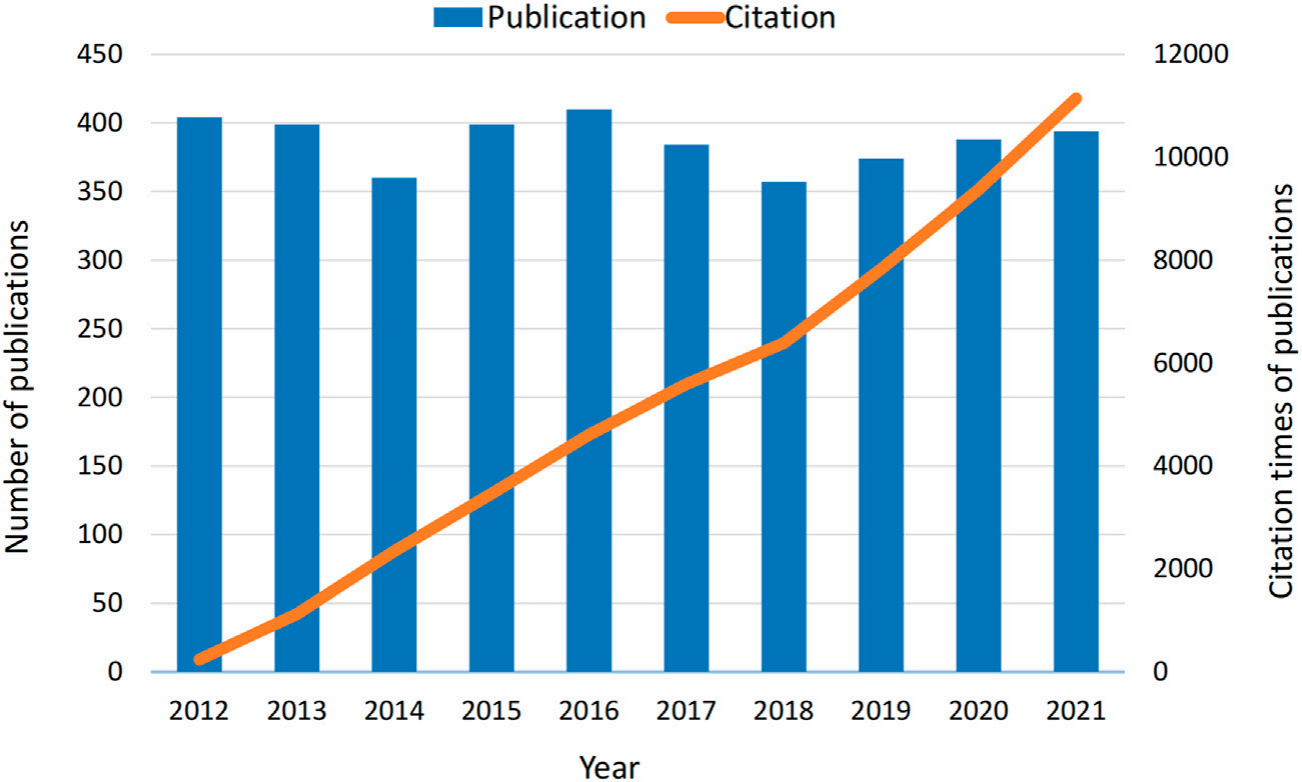
Annual output and citation times of publications with diazepam content from 2012 to 2021.

According to the bibliographic search strategy, a total of 3,870 papers were included in the bibliometric analysis. The number of articles published in each period reflects trends in research in this field. As shown in Figure 2, the number of papers and citations of diazepam articles from 2012 to 2021 remained high, indicating that the study of diazepam has been valued in the past decade.

### 3.2 Countries/regions

**FIGURE 3:**
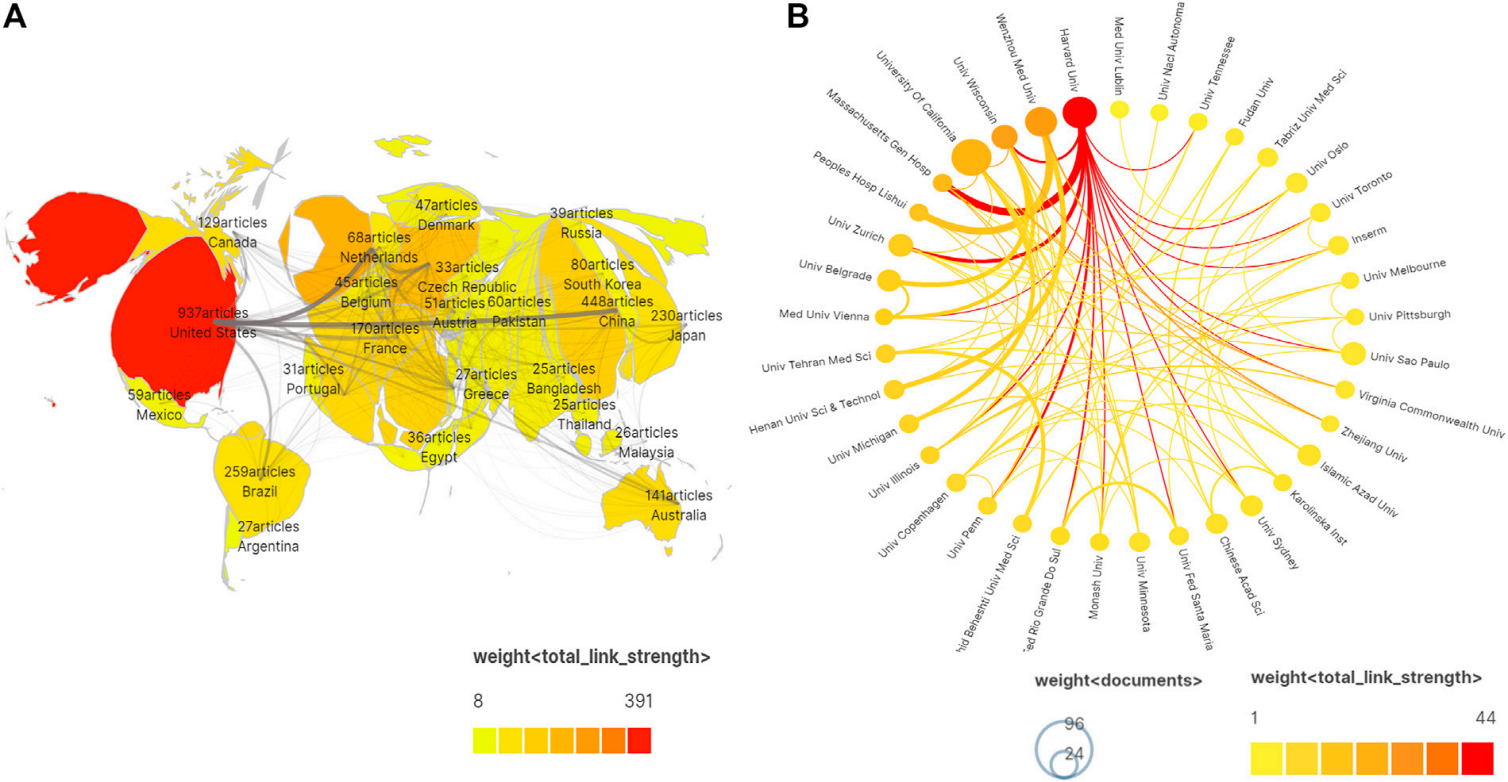
**(A)** Distribution of publications according to country/region. **(B)** Cooperation network among institutions. Note: the thickness of the lines between countries/institutions indicates the strength of cooperation.

A total of 3,870 articles were published by 4,144 institutions in 115 countries/regions. In Table 1, the most significant number of publications came from the United States (937, 24.21%) and China (448, 11.58%). Moreover, the United States had the largest total link strength (TLS = 391), indicating that the USA cooperates most closely with other countries. The next two are the United Kingdom (TLS = 217) and Germany (TLS = 182); different degrees of cooperation had also been established among other countries. The research institutions with the greatest number of publications and citations were the University of California (96, 3,446) and Harvard University (70, 2234). Among the top 10 institutions, most belonged to the United States, followed by China, Brazil, Switzerland, Serbia, Iran, and Australia. This finding suggests that all of these countries/institutions may have played a critical role in diazepam research. In Figure 3, the lines between nodes represent cooperation between countries/institutions; denser lines and a deeper shade of color correspond with closer cooperation.

**TABLE 1:** Distribution of publications according to country/region and institution.

Rank	Country	Count(%)	Citations	TLS	Institution (country)	Count	Citations	TLS
1	United States of America	937 (24.21%)	18,139	391	University Of California (United States of America)	96	3,446	21
2	China	448 (11.58%)	4,210	139	Harvard Univ (United States of America)	70	2,234	44
3	Brazil	259 (6.69%)	3,590	91	Wenzhou Med Univ (China)	61	496	27
4	United Kingdom	231 (5.97%)	4,438	217	Univ Wisconsin (United States of America)	41	444	26
5	Japan	230 (5.94%)	2,585	84	Univ Sao Paulo (Brazil)	38	505	5
6	Germany	216 (5.58%)	4,674	182	Univ Zurich (Switzerland)	37	739	14
7	India	213 (5.5%)	2,269	50	Univ Belgrade (Serbia)	34	349	13
8	Italy	189 (4.88%)	3,396	127	Islamic Azad Univ (Iran)	33	330	6
9	France	170 (4.39%)	3,054	112	Univ Sydney (Australia)	30	931	6
10	Iran	169 (4.37%)	1,722	32	Chinese Acad Sci (China)	29	534	7

### 3.3 Co-authors and co-cited authors

**FIGURE 4:**
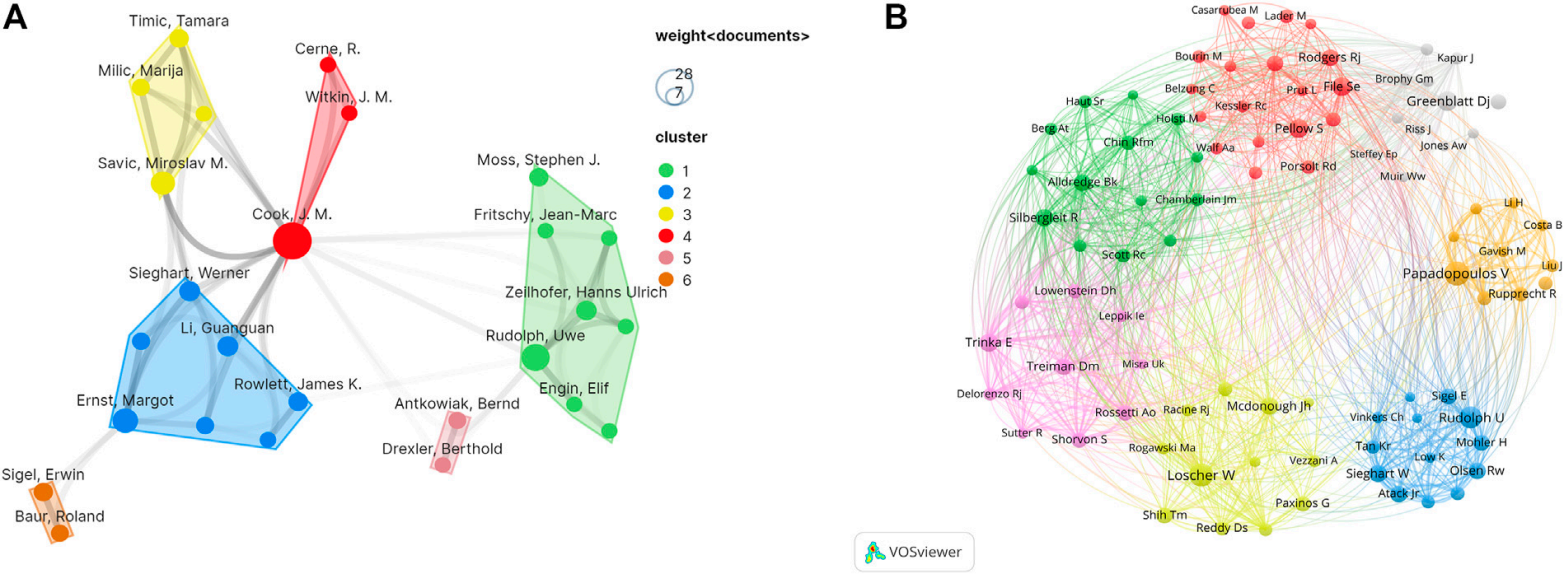
**(A)** VOSviewer visualization map of authors involved in diazepam research. **(B)** VOSviewer visualization map of co-cited authors involved in diazepam research.

In Figure 4A, every node represents a co-author, and the size of the node shows the number of the literature. Then, the lines indicate cooperation between co-authors; darker line shows closer cooperation. Co-cited authors are two or more authors who are cited in other papers at the same time, and therefore have a co-cited relationship. The top ten co-cited authors all have been cited more than 150 times. V Papadopoulos (296) was cited most frequently and had the largest total link strength (TLS = 2630).

In Figure 4B, each node represents a co-cited author, and the size of the node is proportional to the number of citations. The lines between nodes represent cooperation between co-cited authors. As can be seen from Figure 4, the co-authors/co-cited authors were distributed in several different clusters, which show different degrees of cooperation between clusters. A total of 18,326 authors published articles on diazepam. In Table 2, James M Cook had the highest number of published papers (28), followed by Jouyban Abolghasem (18) and Wen Congcong (18). In addition, Rudolph Uwe had the highest citation frequency (539).

**TABLE 2:** Top 10 co-authors and co-cited authors in diazepam research.

Rank	Author	Count	Citations	TLS	Co-cited author	Citations	TLS
1	Cook James M	28	316	62	Papadopoulos V	296	4,529
2	Jouyban Abolghasem	18	191	11	Loscher W	270	2,208
3	Wen Congcong	18	42	53	Rudolph U	235	2,630
4	Rudolph Uwe	15	539	29	Greenblatt Dj	206	1,280
5	Geng Peiwu	14	51	48	Pellow S	187	1,091
6	Loddenkemper Tobias	14	265	13	File Se	186	1,552
7	Tonon Marie-Christine	14	237	70	Trinka E	178	2,960
8	Leprince Jerome	13	217	67	Mcdonough Jh	166	2,443
9	Cloyd James C	12	266	22	Sieghart W	162	1,901
10	Ernst Margot	12	258	25	Silbergleit R	161	2,533

### 3.4 Journals and co-cited academic journals

**FIGURE 5:**
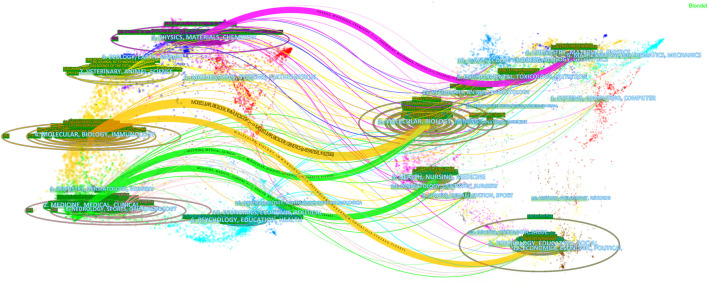
Dual map overlay of journals with diazepam content. Note: the citing journals are on the left, and the cited journals are on the right. Color paths denote reference relationships, and thick lines denote main paths.

We also performed a visual analysis of the journals that had published articles on diazepam using the VOSviewer software. And, we found that the 3,870 articles related to diazepam were published in 1,133 academic journals. Table 3 shows that the top three journals, by output, are Epilepsia (60, 1.55%), the Latin American Journal of Pharmacy (60, 1.55%), and Neuropharmacology (58, 1.50%). Diazepam-related literature mainly focused on three areas: epilepsy, psychotropic drugs, and pharmacology. Furthermore, in Table 3 that most of the journals belong to Q1/Q2. The co-cited times a journal can reflect the influence of the journal in a particular field of study. Among the co-cited journals, the top 10 journals were cited over 1,000 times. Epilepsia (3,424) was the most frequently cited journal, followed by J Neurosci (2,884), Psychopharmacology (2,287), and Pharmacology Biochemistry & Behavior (2,253). According to the 2020 JCR, most of the co-cited journals among the top-ranked journals belong to Q1/Q2. The distribution of relationships between journals can be shown by a dual map overlay of journals. In Figure 5, the cited journals are shown on the left and the cited journals on the right. The colored paths between the two sides indicate the cited relationships. There are five main citation paths, including two green, two orange, and one purple path. The purple path indicates that studies published in Chemistry/Materials/Physics journals are cited in studies in Physics/Materials/Chemistry. The two orange paths indicate that studies published in Molecular/Biology journals and Psychology/Education/Social journals are cited in studies in Molecular/Biology/Immunology journals. And, the green paths indicate that studies published in Molecular/Biology journals and Health/Nursing/Medicine journals are generally cited by Medicine/Medical/Clinical journals.

**TABLE 3:** Top 10 journals and co-cited journals publishing diazepam research articles. IF, Impact Factor; JCR, Journal Citation Reports.

Rank	Journal	Count (%)	IF (2021)	JCR	Co-cited journal	Citation	IF (2021)	JCR
1	Epilepsia	60 (1.55%)	6.740	Q1	Epilepsia	3,424	6.740	Q1
2	Latin American Journal of Pharmacy	60 (1.55%)	0.229	Q4	J Neurosci	2,884	6.709	Q1
3	Neuropharmacology	58 (1.50%)	5.273	Q1/Q2	Psychopharmacology	2,287	4.415	Q2
4	Behavioural Brain Research	52 (1.34%)	3.352	Q2	Pharmacol Biochem Be	2,253	3.697	Q2/Q3
5	Plos One	52 (1.34%)	3.752	Q1	Eur J Pharmacol	1,768	5.195	Q1
6	Epilepsy & Behavior	50 (1.29%)	3.337	Q2/Q3	P Natl Acad Sci Usa	1,758	12.77	Q1
7	Pharmacology Biochemistry and Behavior	40 (1.03%)	3.697	Q2/Q3	J Pharmacol Exp Ther	1,731	4.441	Q1
8	Journal of Ethnopharmacology	39 (1.01%)	5.195	Q1	Neuropharmacology	1,627	5.273	Q1
9	Psychopharmacology	37 (0.96%)	4.415	Q2	Brain Res	1,583	3.610	Q3
10	Forensic science international	35 (0.90%)	2.676	Q2	Behav Brain Res	1,309	3.352	Q2

### 3.5 Co-cited references and reference bursts

**FIGURE 6:**
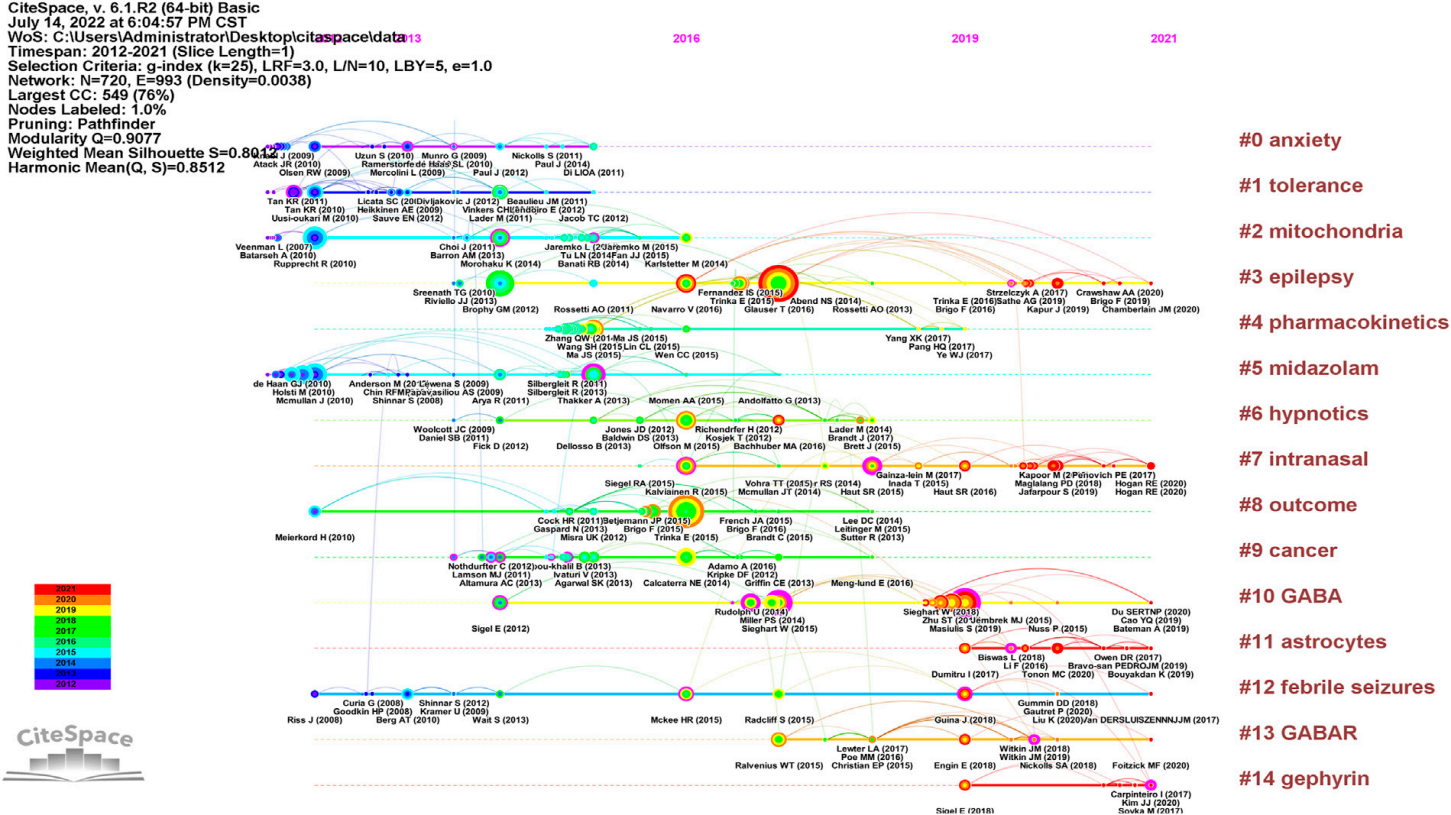
Timeline of diazepam research references. Note: the size of the nodes is positively correlated with the frequency of citation, the curve between nodes represents the citation relationship, the straight line represents duration, the color of the lines and nodes corresponds to the color of the year.

**FIGURE 7:**
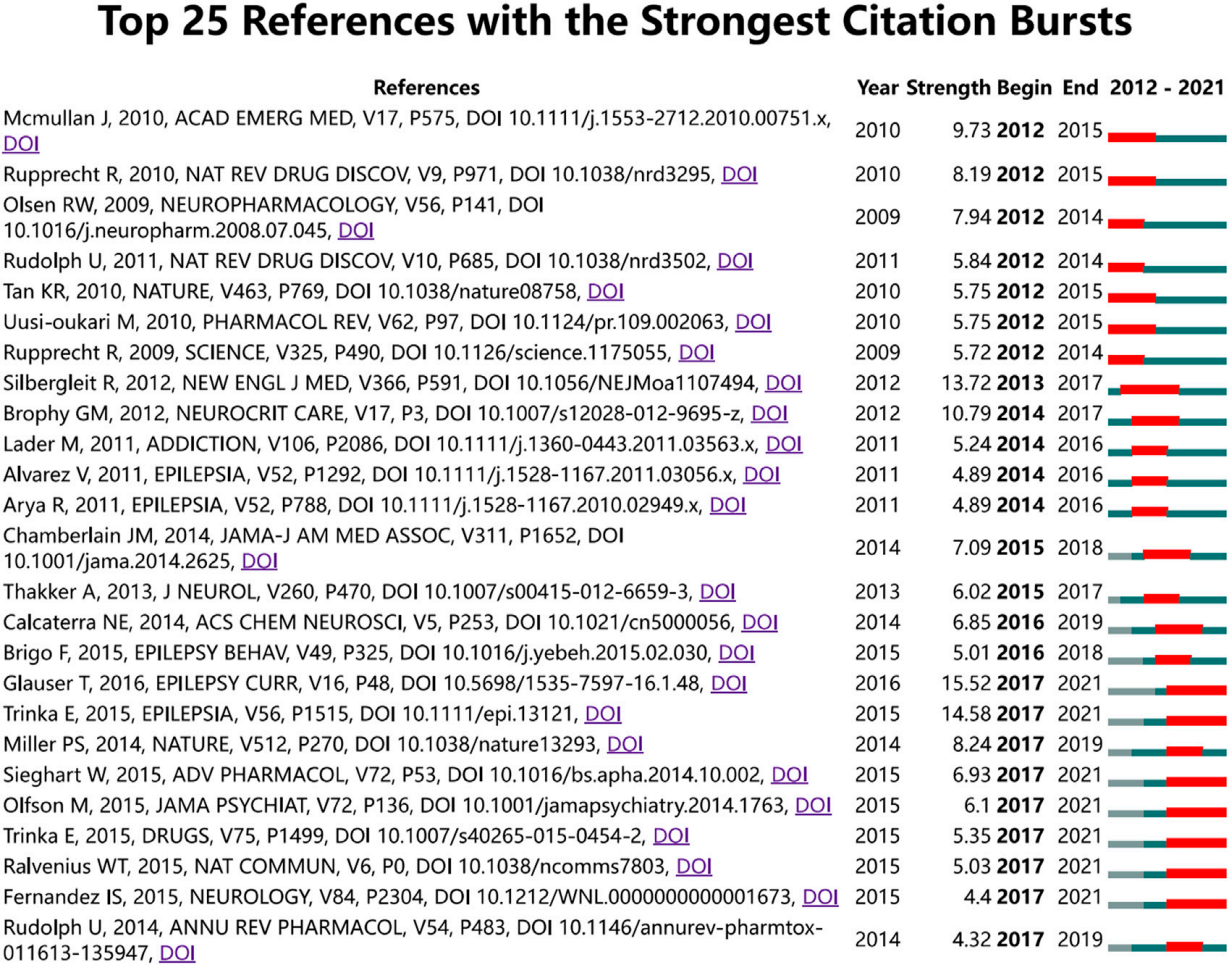
CiteSpace map of the 25 references of diazepam research with the strongest citation bursts. Note: the light blue bars reflect the period, the sky-blue bars reflect the publication period, and the red bars reflect the duration of the burst.

The timeline visualization presents a temporal overview of nodes, links, and clusters. As illustrated in Figure 6, the references selected in this study were mainly clustered in 14 categories: anxiety, tolerance, mitochondria, epilepsy, pharmacokinetics, midazolam, hypnotics, intranasal formulation, outcomes, cancer, gamma-aminobutyric acid (GABA), astrocytes, febrile seizures, GABAR, and gephyrin. The research hotspots and fields of concern differ in different periods. Anxiety, tolerance, mitochondria, midazolam, outcomes, GABA, and febrile seizures were earlier areas of concern. Over time, the research foci of epilepsy, intranasal formulations, GABA/GABAR, astrocytes, febrile seizures, and gephyrin in the field of diazepam has gradually become prominent. Two articles or more articles are cited by one or more papers at the same time, then these two articles are in a co-citation relationship. Among the retrieved co-cited references, Table 4 shows the top 10 co-cited references and the references with citation bursts, of which the top three for both co-cited reference counts and references with citation bursts are Silbergleit R (81, 13.72), Glauser T (73, 15.52), and Trinka E (59, 14.58). Moreover, the different references exhibit different bursts in different periods. As can be seen in Figure 7, the bursts of these three references have the longest duration (2015–2021), which reflects the current research focus to some extent and shows an important consultative value. Analysis of Table 4 and Figure 7, shows that the top 10 cited publications, other than one article (Masiulis et al., 2019), were all included in the references with the strongest citation bursts. These literature can be considered the most valuable and influential research in the field. First, the most highly cited study published in the New England Journal of Medicine (IF = 176.079) showed that the use of intramuscular midazolam, rather than an intravenous agent, in the hospital setting, compared favorably with intravenous diazepam in the emergency treatment of status epilepticus, with sustained convulsions for more than 5 min (Silbergleit et al., 2012). Second, the strongest citation burst came from an article published by Tracy Glauser, MD (2017–2021). In 2016, the American Epilepsy Society (AES) published clinical guidelines for adults and children with convulsive status Epilepticus (CSE) in which the use of intravenous diazepam is Level A evidence. Third, the basis of the pharmacological regulation mechanism of the gamma-aminobutyric acid type A (GABAA) receptor is still largely unknown. As shown in Table 4, an article published in Nature in 2019 with high centrality, used electron cryo-microscopy to describe diazepam’s binding mode and mechanism to its receptor, which lays a foundation for further understanding of diazepam on heteromeric synaptic GABAAR (Masiulis et al., 2019). It is believed that there will be a higher number of citations as time goes by. One of the articles on the use of benzodiazepines was published in JAMA Psychiatry. This was a retrospective, descriptive analysis of prescriptions for benzodiazepines. It indicated that despite caution concerning the risks associated with long-term benzodiazepine use, long-term benzodiazepine use remains common among older patients. (Olfson et al., 2015). From this, we can speculate on the status of diazepam use.

**TABLE 4:** Top 10 co-cited references and references with citation bursts related to diazepam.

Rank	References citation	Journal	Count	Year	Centrality	Bursts	Citations
1	Intramuscular versus intravenous therapy for prehospital status epilepticus	N Engl J Med	81	2012	0.09	13.72	391
2	Evidence-Based Guideline: Treatment of Convulsive Status Epilepticus in Children and Adults: Report of the Guideline Committee of the American Epilepsy Society	Epilepsy Curr	73	2016	0.10	15.52	497
3	A definition and classification of status epilepticus--Report of the ILAE Task Force on Classification of Status Epilepticus	Epilepsia	59	2015	0.07	14.58	881
4	Guidelines for the evaluation and management of status epilepticus	Neurocrit Care	40	2012	0.01	10.79	843
5	Midazolam versus diazepam for the treatment of status epilepticus in children and young adults: a meta-analysis	Acad Emerg Med	32	2010	0.03	9.730	115
6	Beyond classical benzodiazepines: novel therapeutic potential of GABAA receptor subtypes	Nat Rev Drug Discov	29	2011	0.03	8.19	450
7	GABA(A) receptor signalling mechanisms revealed by structural pharmacology	Nature	28	2019	0.20	10.49	216
8	Translocator protein (18 kDa) (TSPO) as a therapeutic target for neurological and psychiatric disorders	Nat Rev Drug Discov	27	2010	0.04	8.19	632
9	Lorazepam vs. diazepam for pediatric status epilepticus: a randomized clinical trial	JAMA	26	2014	0.05	7.09	105
10	Benzodiazepine use in the United States	JAMA Psychiat	26	2015	0.04	6.1	404

### 3.6 Keywords

**FIGURE 8:**
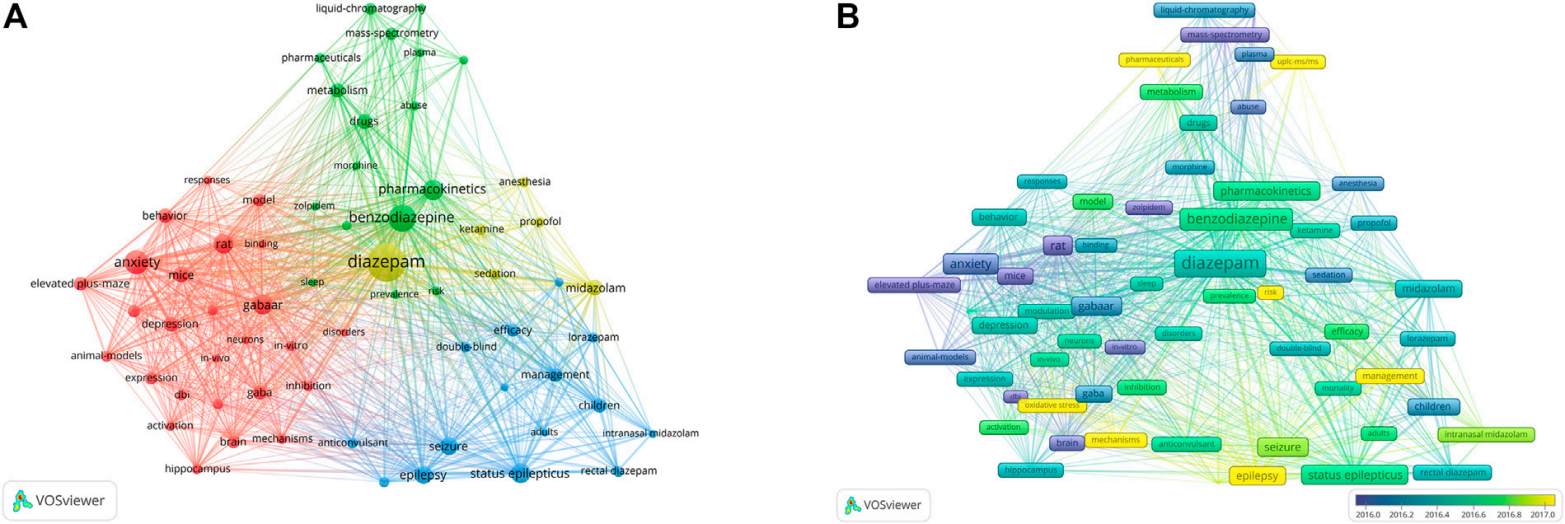
**(A)** Keyword co-occurrence analysis. Closely related keywords are clustered by color, and clusters of four colors (red, blue, green, yellow) represent four clusters. **(B)** Overlay visualization map of keyword co-occurrence analysis. Nodes marked in purple or blue indicate keywords that appear relatively early, while keywords marked in yellow indicate current research foci.

Keywords provide an overview of the research topic and an understanding of the hot topics of research in a particular field. In this study, keyword co-occurrence network graphs were constructed using VOSviewer. In all the literature, we extracted keywords that appeared at least 60 times. Information on the 22 most frequently occurring keywords is summarized in Table 5. Other than diazepam, the high-frequency keywords in this study were benzodiazepine, anxiety, status epilepticus, GABAAR, pharmacokinetics, seizure, epilepsy, midazolam, children, depression, behavior, metabolism, GABA, management, elevated plus-maze, brain, efficacy, stress, mass-spectrometry, liquid-chromatography, rectal diazepam, and sedation. As shown in Figure 8A, Network diagrams that are clustered can reflect the basic knowledge structure of the research area, and most were clustered into four categories: the mechanism of action of diazepam (GABAR), application in disease (including status epilepticus, anxiety, *etc.*), pharmacokinetics, and the assessment and management of diazepam; each cluster represents relatively large territories of diazepam research, indicating that these were significant topics in the study of diazepam. In addition, in an overlay visualization map of the keyword co-occurrence analysis graph shown in Figure 8B, pharmacokinetics, risk, UPLC-MS/MS, management, oxidative stress, mechanisms, and epilepsy represent the current research foci, which may reflect future trends in diazepam research.

**TABLE 5:** Top 22 keywords related to diazepam.

Rank	Keyword	Count	TLS	Rank	Keyword	Count	TLS
1	benzodiazepine	648	1,802	12	metabolism	179	465
2	anxiety	485	1,475	13	gaba	175	569
3	status epilepticus	345	1,283	14	management	166	536
4	gabaar	338	1,071	15	elevated plus-maze	157	513
5	pharmacokinetics	375	1,050	16	brain	139	449
6	seizure	268	992	17	efficacy	136	471
7	epilepsy	284	979	18	stress	131	438
8	midazolam	213	791	19	mass-spectrometry	128	315
9	children	187	663	20	liquid-chromatography	126	318
10	depression	193	584	21	rectal diazepam	109	422
11	behavior	180	601	22	sedation	101	296

## 4 Discussion

### 4.1 General information

The trends in the number of publications and article citations reflect the development rate and research progress of a field. As can be seen in Figure 2, the number of articles and citations on diazepam are generally at a relatively high level, indicating that diazepam has been an important research direction in recent years and has an excellent development trend in the future.

According to the distribution of countries/regions, the USA is the most productive country, suggesting that it is a leading country in diazepam research. Concurrently, it has the highest TLS, which means that the United States has the closest cooperation with other countries and even plays a significant bridging role in global cooperation. Among the top 10 institutions, three were from the United States and two were from China, which also reflects the reason that the two countries have the largest number of publications, from another aspect. Although there are different degrees of cooperation between countries, the TLS of cooperation among some of the top 10 institutions can be improved. Therefore, we hope that countries and institutions, especially the top ones, can cooperate even further. The publication count of James M Cook, from the United States, ranked first in the author list, and had a high TLS; Rudolph had the highest number of citations, indicating great academic influence and outstanding scientific achievement in diazepam research in the past decade. The citation count and TLS of V Papadopoulos ranked first in co-cited authors, reflecting his strong academic influence on diazepam research. Of the published and cited journals, Epilepsia ranked first with the highest number of publications and citations of diazepam research. Although diazepam has been used in epilepsy for nearly 70 years, it is still an important topic in epilepsy therapy, especially status epilepticus. Furthermore, both pharmacology and behavior are also important directions.

### 4.2 Hotspots and frontiers

From the clustering of references, reference bursts, frequent keywords, keyword clustering, and keyword hotspot changes, it can be seen that pharmacokinetics; the mechanism of action of diazepam (GABA/GABAR, gephyrin, astrocytes, and oxidative stress); its application in disease (especially in status epilepticus, intranasal use, and febrile seizures); and the risk, testing, and management of diazepam are the four directions of research focus and future development.

#### 4.2.1 Pharmacokinetics of diazepam

Diazepam is a medium potency long-acting drug with high bioavailability, which is completely and rapidly absorbed 0.5–1.5 h after oral administration, while the absorption of intramuscular administration is related to the injection site, depth, and amount of adipose tissue. Plasma concentration peaks within minutes of intravenous administration. With long-term administration, steady-state levels are reached after 5–14 days (Altamura et al., 2013). Diazepam is mainly metabolized in the liver through CYP3A4, and diazepam and its metabolites are excreted mainly in the urine. The plasma half-life is 24–48 h and shows individual differences, especially in the elderly population. The half-life of diazepam increases after a drug overdose, with significant prolongation of the half-life of diazepam and its metabolites, and drug accumulation, leading to adverse drug reactions (Kienitz et al., 2022).

#### 4.2.2 Mechanism of action of diazepam

##### 4.2.2.1 GABAAR and diazepam

The pharmacological effects of diazepam are mediated through two main types of receptors: the GABAA receptor and the transporter protein (TSPO). Diazepam is a targeted drug acting on the GABAA receptor, a positive variant modulator. The GABAA receptor is a pentamer composed of three subunits of αβγ in a 2:2:1 ratio, arranged counterclockwise and assembled into a ring-like pentameric complex with a chloride channel in the middle. Diazepam acts on GABAA receptors, enhances the action of GABA and GABAA receptors, regulates the opening of chloride channels, increases the entry of Cl-into the cell, and causes hyperpolarization and inhibitory postsynaptic potentials in the cell membrane. Diazepam acts on the alpha subunit of the GABAA receptor, which includes six subtypes (α1-6). According to the different affinities of benzodiazepines binding to GABAAR, they are divided into sensitive and insensitive GABAAR. GABAAR containing α1, α2, α3, and α5 subunits are sensitive, while α4 and α6 subunits belong to insensitive GABAAR. This is because the replacement of histidine with arginine in the α4 and α6 subunits causes it to lose its affinity (McKernan et al., 2000). Different subtypes of GABAR play different roles in the central nervous system. GABAARs containing the α1 subunit mediate sedative, cis-amnesic, and partially, anticonvulsant effects. GABAARs containing the α2 subunit mediate anxiolytic and, to a large extent, myorelaxant effects. GABAARs containing α3 and α5 subunits also contribute to myorelaxant effects. GABAAR containing the α2 and α5 subtypes are the most promising targets for addressing schizophrenia symptoms. GABAARs expressing the α5 subunit were shown to modulate the spatiotemporal memory of benzodiazepines, associated with addiction (Tan et al., 2011; Rudolph and Möhler, 2014; Rudolph and Knoflach, 2011). The beta subunit is associated with sedative, ataxic, and narcotic drug effects (Gee et al., 2010; Sigel and Ernst, 2018). Diazepam is not selective for multiple subunits of the GABAAR and therefore has different effects and side effects. Additionally, Shisa7 is an auxiliary subunit of GABAAR, and endogenous Shisa7 co-localizes with gephyrin, interacts with GABAARs, regulates GABAAR transport to synapses, and modulates channel dynamics and pharmacology. Shisa7 was found to enhance diazepam-induced currents in the α2β3γ2/Shisa7 complex and to play a key role in regulating tonic inhibition in hippocampal neurons (Han et al., 2019; Wu et al., 2021). With deeper understanding of the mechanism of action of diazepam and the receptors, a variety of drugs targeting the GABAAR subunits have been developed in recent years, although the associated side effects were not resolved (Cerne et al., 2022). Some new subunit-specific GABAA receptor modulators that may have new indications, such as analgesia, schizophrenia, cognitive enhancement, depression, and stroke have been developed (Rudolph and Knoflach, 2011). The mechanism of action of diazepam is largely unknown, and thanks to the emerging technological developments in electron cryo-microscopy, the current introduction of high-resolution electron cryo-microscopy has revealed the molecular basis of diazepam-regulated receptors, which is important for the understanding of GABAAR biology and the development of diazepam pharmacology. The search for new and effective targets and drug development is of great significance (Zhu et al., 2018; Masiulis et al., 2019; Masiulis et al., 2019; Scott and Aricescu, 2019; García-Nafría and Tate, 2020; Kim et al., 2020).

##### 4.2.2.2 TSPO and diazepam

Diazepam acts not only on the GABA(A) receptors but also on the TSPO (Rupprecht et al., 2009). TSPO, formerly known as peripheral-type benzodiazepine receptor, is a universal protein located primarily on the outer membrane of mitochondria. It can transport cholesterol through mitochondrial membranes and act on lipid metabolism, but its exact physiological role is still controversial. This protein was also later found in the central nervous system and is mainly expressed in microglia, reactive glia, and neurons. Studies have shown that TSPO expression may be a biomarker of brain inflammation and reactive gliosis. The expression of TSPO increased in brain injury and neuroinflammation, and decreases in patients with anxiety, schizophrenia, and major depression (Guilarte, 2019; Barresi et al., 2021). In addition, various TSPO ligands are being developed as neuroimaging agents, and although there are some problems with side effects, preliminary clinical trials show that they may have potential in diagnosing and treating mental disorders, such as neurological disorders and anxiety (Rupprecht et al., 2010). In a recent study on diazepam published in Nature Neuroscience, in TSPO^−/−^ mice, regardless of the dose and timing of diazepam administration, there was no effect on the density and formation of dendritic spines or the number and morphology of microglia in the mouse brain. However, long-term use of diazepam can enhance the phagocytosis of microglia by TSPO, induce dendritic spines, and damage cognitive function. The damage caused by high-dose diazepam is more obvious (Shi et al., 2022).

##### 4.2.2.3 Gephyrin and diazepam

In inhibitory synapses, the Gephyrin is one of the most studied proteins, belongs to the core scaffolding proteins, and plays an important role in synaptic anchoring with GABAR subtypes (Hoffmann and Milovanovic, 2021). Gephyrin participates in the anchoring of GABAARs in the postsynaptic region, affects the aggregation and diffusion of synaptic GABAARs, regulates the number of gephyrins, and regulates inhibitory synaptic plasticity (Bai et al., 2021; Brockbals et al., 2021; Hoffmann and Milovanovic, 2021). Gephyrin and GABAA are interdependent, studies have shown that knocking out gephyrin will destroy GABAAR clusters without affecting their overall protein levels. The postsynaptic clusters of GABAAR and gephyrin were almost completely lost by various GABAAR subunit knockout models (Essrich et al., 1998). Gephyrin is closely related to epilepsy, autism, schizophrenia, and other neuropsychiatric disorders (Barberis, 2020). Research shows that, during epilepsy (incubation period), the levels of total gephyrin and GABAAR subunits γ2, α2/3, and α4 protein in the hippocampus of animals decreased significantly, and gephyrin knockout mice died in a state similar to status epilepticus within 24 h of birth (Groeneweg et al., 2018). The interaction between GABAAR and gephyrin is controlled by palmitoylation. DHHC12 is a palmitoyltransferase, which specifically palmitoylates gephyrin, promotes the localization of synaptic gephyrin, and affects the conformational characteristics of GABAAR. The lack of gephyrin palmitoylation reduces the GABAAR function of the basolateral amygdala and causes anxiety (Dejanovic et al., 2014). Recent studies have shown that diazepam can activate DHHC12 to increase the palmitoylation of gephyrin in the basolateral amygdala, rapidly reduce lateral diffusion of GABAAR, increase synaptic stability and aggregation, and play an anti-anxiety role. Additionally, the knockout of DHHC12 almost eliminates the anti-anxiety effect of diazepam (Shen et al., 2019).

##### 4.2.2.4 DBI and diazepam

In 1983, Guidotti et alfound that the brain contains competitive [^3^H] diazepam binding inhibitor, which can replace the benzodiazepine receptor in GABAA. Intraventricular administration will induce conflict and anxiety behavior, so it is called a diazepam binding inhibitor(DBI) (Guidotti et al., 1983; Tonon et al., 2020). In the study of DBI, researchers found that it is a peptide of the network peptide family, which is produced by astrocytes, regulates energy metabolism, and inhibits apoptosis caused by oxidative stress and acts as a neuronal protector (Ghouili et al., 2018; Masmoudi-Kouki et al., 2020). However, there are very few studies on astrocytes and oxidative stress related to diazepam.

Diazepam acts on multiple receptors, of which the GABAAR is complex and the specific mechanism of action between them is not known. Future studies will further delve into the mechanism of action and the related ligands of diazepam to find new effective targets and facilitate the development of more effective drugs for neurological diseases and psychiatry (Sigel and Ernst, 2018; Han et al., 2019; Han et al., 2021).

#### 4.2.3 Hotspots and frontiers in the clinical use of diazepam

Notably, by analyzing the references, it appears that articles related to status epilepticus have the highest number of citations and the strongest citation bursts, while febrile seizures are also a current hot spot.

Status epilepticus (SE) is a life-threatening condition with an acute onset that requires prompt treatment. Benzodiazepines are the mainstay of emergency treatment of epilepsy because of their rapid onset of action, excellent efficacy, and well-tolerated. Diazepam, midazolam, and lorazepam are included in the American Epilepsy Association guidelines as first-line treatments for persistent epilepsy (Guterman et al., 2021). Possible drowsiness, as well as rare side effects such as respiratory depression and hypoxia, should be noted during use. Diazepam is available in many ways, including oral, intravenous, direct, and intranasal. Establishing peripheral venous catheters during poor venous status or seizures or outside the hospital may result in delayed drug administration. (Kienitz et al., 2022). Rectal diazepam licensed in the United States in 1997. However, liver metabolism, absorption variability, and taking medicine in public places causes embarrassment to patients. While trans-nasal diazepam is a new form of administration, after rigorous testing, the FDA recently approved the intranasal form of diazepam (Valtoco^®^) for the acute treatment of frequent, intermittent, and stereotypic seizures, i.e., seizure clusters or acute repetitive seizures, in patients 6 years of age and older (Segal et al., 2021). The advantages of intranasal administration are as follows: first, the nasal mucosa has a rich vascular network, drug absorption is faster, and epileptic seizure inhibition is faster. Additionally, trans-nasal administration avoids liver metabolism, and pharmacokinetic variability is low. In addition, trans-nasal administration does not need the cooperation of patients, and is therefore easier to use and more easily accepted and managed by society (Hogan et al., 2020a; Higdon and Sperling, 2021). The safety assessment of long-term use of the diazepam nasal spray showed that it is safe, well tolerated, generally causes less discomfort, and has no serious adverse reactions (Hogan et al., 2020a; Hogan et al., 2020b; Wheless et al., 2021). In a long-term safety analysis of repeated nasal diazepam in pediatric patients (6–17 years of age) with frequent seizures showed a safety profile consistent with that of all study populations (ages 6–65 years). (Tarquinio et al., 2022). Hence, intra-nasal first aid is a good treatment option.

The most frequent type of seizures in children is febrile seizures (FS), which have a cumulative incidence of 2%–5% in Western Europe and the United States and recur in 30% of cases. (Kubota et al., 2020). Most patients with FS do not require conventional antiepileptic treatment due to its self-limitation and favorable prognosis. However, prolonged FS seizures need to be prevented from evolving into febrile status epilepticus (FSE) and to reduce adverse consequences (Hesdorffer et al., 2016). Diazepam is used as a treatment for FS to enhance GABA-mediated inhibition of neuronal activity, which acts as a seizure suppressor (Natsume et al., 2017). In a study of children with FS for the first time, patients who used both diazepam and acetaminophen had fewer recurrences than those who used diazepam alone (Tanaka et al., 2022). The prophylactic use of diazepam has been controversial. In a retrospective study in Japan comparing rectal administration and seizure recurrence within 24 h of the first seizure, in 2011–2015 (when prophylactic diazepam use was recommended before guideline revision) and in 2016–2018 (when routine diazepam use was not recommended after guideline revision), results showed an association between increased FS recurrence within 24 h and decreased prophylactic diazepam use (Inoue et al., 2020). However, a recent meta-analysis showed that for children at increased risk of frequent or complex seizures, although intermittent diazepam and continuous phenobarbital may reduce the recurrence rate, the adverse effects are up to 30%; therefore, intermittent and continuous treatment with antiepileptic drugs is not recommended and families and parents should receive sufficient information about the disease (Offringa et al., 2021).

#### 4.2.4 Risks of diazepam

Diazepam is widely used and effective, but there are risks that are gaining attention, such as abuse, addiction, and long-term irrational use of diazepam in the elderly with memory impairment, falls, fractures, excessive sedation, cognitive impairment, respiratory depression, and even death from concomitant use with opioids.

First, the abuse of benzodiazepines is a global public health problem. Benzodiazepines and other sedatives are the third largest illicit or prescription drugs in the United States, and the global abuse rate seems to be similar to that of the United States. Diazepam is the most widely used benzodiazepine drug in some countries. Although it is safer, benzodiazepine overdose can result in coma and death (Chan et al., 2020; Lopez et al., 2021). A systematic review of human studies that examined the prevalence, patterns, correlations, causes, motivations, sources, and effects of benzodiazepine abuse was published. (Votaw et al., 2019). These findings will help to understand and deal with drug abuse problems. However, more research is needed on the epidemiology of drug abuse. In recent years, for the *in vivo* determination of numerous medicines, UPLC-MS/MS has gained popularity. UPLC-MS/MS is characterized by rapidity, specificity, sensitivity, and accuracy, and plays an important role in drug confirmation and quantification (Qi and Liu, 2021). Diazepam can be abused for entertainment or in other situations. Determination of diazepam levels in whole blood, serum, plasma, oral samples hair, and urine by UPLC-MS/MS is helpful to study of the pharmacokinetics of diazepam and has forensic significance (Laloup et al., 2007; Wang et al., 2017; Brockbals et al., 2021).

The use of benzodiazepines carries several hazards, and most guidelines or expert consensus recommend short-term use, but long-term use of benzodiazepines is still widespread, especially in the elderly, mainly for insomnia and anxiety (Gerlach et al., 2018a; Gerlach et al., 2018b; Delaš Aždajić et al., 2021). Short-term use of the drug can cause memory loss, however, there is a great deal of concern about whether long-term use can lead to dementia (Bocti et al., 2012). There is substantial evidence that long-term use of benzodiazepines can increase the risk of dementia (de Gage et al., 2012; de Gage et al., 2014; Pariente et al., 2016; Shash et al., 2016). Paradoxically, there is rising clinical evidence that benzodiazepines are not linked to dementia or cognitive decline and may even have neuroprotective effects. The possible reason for this is that symptoms such as insomnia, anxiety, and depression may occur before dementia is detected, and benzodiazepines are used in the treatment of dementia’s early symptoms. (Gray et al., 2016; Davies et al., 2017; Pilipenko et al., 2019; Osler and Jørgensen, 2020; Rosenbaum, 2020). In animal experiments, very low and moderate doses of diazepam (2 weeks) were found to modulate early pathological events, by reduction of neuroinflammation, astro- and micro-gliosis, and increase in GABAergic and glutamatergic activity in a model of sporadic Alzheimer’s disease (AD), acting to improve memory (Pilipenko et al., 2018; Pilipenko et al., 2019). In the hereditary form of recessive familial AD models, the use of moderate doses of diazepam (4 weeks) can prevent amyloid β oligomer accumulation (Umeda et al., 2017). Even so, close attention needs to be paid to benzodiazepine prescription in the elderly, and further research is needed on the specific mechanisms of diazepam dose and duration of use on cognition and the pros and cons of its use.

Overall, risks and drug management should be taken into account in the use of diazepam, and effective protocols should be developed for specific patient categories (Hayhoe and Lee-Davey, 2018; Hirschtritt et al., 2021).

## 5 Limitations

This study has several limitations. First, VOSviewer and CiteSpace mainly perform quantitative analysis of terms in literature, which cannot completely replace the systematic review. When different clustering and calculation methods are chosen the results will be slightly different. Due to the limitations of the software, some emerging fields or rare diseases with less research may not be displayed. High-quality articles that have just been published may have less of an impact because they have not had enough time to gather enough citations. Second, just a small fraction of the literature from other databases has been included in the literature we chose because it is only based on WoSCC. However, it is generally acknowledged that WoSCC is the best database for bibliometric analysis and that it should be used; data from this database can provide the majority of the information about a particular topic (Cheng et al., 2022). Finally, because only English publications were chosen and non-English publications were omitted, the study may have been biased toward nations that publish in international journals where English is the primary language due to the value of non-English articles being undervalued. In the early stages of this study we analyzed the terms “Apaurin,” “Diapam,” “Diazepamum,” “Valium,” and found 40 articles. All these articles referenced diazepam, had very few citations, or contained uncorrelated studies. We believe that the removal of this literature would not have an effect on the conclusion, and the results will not change much. But there is little doubt that the visual analysis, which was based on the literature, provides a solid framework for comprehending diazepam study.

## 6 Conclusion

This study performed a comprehensive bibliometric analysis of diazepam between 2012 and 2021. Our findings suggest that, whether in the number of publications or the academic level, the United States occupies a leading position. Of all the authors and co-cited authors, James Cook, Rudolph Uwe, and V Papadopoulos contributed the most. Researchers in the future should improve international collaboration. Most of the diazepam-related articles were published or cited in globally renowned journals, which suggests that it has attracted a lot of interest. Currently, the research on diazepam primary focuses on status epilepticus, GABAR, pharmacokinetics, intranasal formulations, gephyrin, risk, management, and UPLC-MS/MS, which may also be the focus of future research.

## Data Availability

The raw data supporting the conclusion of this article will be made available by the authors, without undue reservation.
